# Predictor factors for non-invasive mechanical ventilation failure in severe COVID-19 patients in the intensive care unit: a single-center retrospective study

**DOI:** 10.1186/s44158-022-00038-7

**Published:** 2022-02-15

**Authors:** Antonio Romanelli, Pietro Toigo, Giuliana Scarpati, Angela Caccavale, Gianluigi Lauro, Daniela Baldassarre, Filomena Oliva, Graziella Lacava, Gabriele Pascale, Ornella Piazza

**Affiliations:** 1 Department of Anaesthesia and Intensive Care, AOU “San Giovanni di Dio e Ruggi DʼAragona”, Salerno, Italy; 2 Department of Medicine and Surgery, Università Degli Studi di Salerno, Baronissi, Italy

**Keywords:** COVID-19, ARDS, Non-invasive mechanical ventilation, Intensive care unit, Predictive factor

## Abstract

**Background:**

During the COVID-19 pandemia, non-invasive mechanical ventilation (NIV) has been largely applied. Few data are available about predictors of NIV failure in critical COVID-19 patients admitted to ICU. The aim of this study is to analyze clinical and laboratory features able to predict non-invasive ventilation success in avoiding endotracheal intubation.

**Methods:**

A retrospective observational study was performed in our COVID-19 ICU during a 6-month period. Demographic, clinical, laboratory, imaging, and outcome data were extracted from electronic and paper medical records and anonymously collected.

**Results:**

Eighty-two severe COVID-19 patients were supported by NIV at ICU admission. The median PaO_2_/FiO_2_ ratio was 125 [98.5–177.7]. NIV failed in 44 cases (53%). Patients who experienced NIV failure had a higher Charlson Comorbidity Index (median value 4) compared to those who were dismissed without endotracheal intubation (median 2, *p* < 0.0001). At Cox regression analysis, the Charlson Comorbidity Index represented a predictive factor related to NIV failure. PaO_2_/FiO_2_, CPK, INR, and AT III at ICU admission showed a significant relationship with the outcome, when single variables were adjusted for the Charlson Comorbidity Index.

**Conclusion:**

The Charlson Comorbidity Index may be helpful to stratify patients’ risk of NIV failure in a severe COVID-19 population; even if this study, retrospective design does not allow definitive conclusions.

**Supplementary Information:**

The online version contains supplementary material available at 10.1186/s44158-022-00038-7.

## Background

The most relevant clinical manifestation of COVID-19 [1] is the development of interstitial pneumonia, evolving in about 5–15% of cases to acute respiratory distress syndrome (ARDS), requiring admission in intensive care [2]. Older age and the presence of multimorbidities are related to a higher risk of mortality, and the Charlson Comorbidity Index, which is used by geriatricians to predict 10 years of mortality, can predict COVID-19 mortality with an exponential increase in the odds ratio by each point of score [3].

Chinese [4], British [5], American [6], and Australian [7] guidelines recommended safe endotracheal intubation and initiation of invasive mechanical ventilation in critically ill COVID-19 patients. Two objectives justified this approach: (a) to reduce the airborne dispersion of viral particles, preventing contagion between healthcare personnel [8]; (b) avoid the onset of sudden cardiac arrest and death in patients with severe hypoxia [9]. Although experts support the use of early endotracheal intubation to prevent “self-inflicted lung injury” (SILI) [10], a meta-analysis suggests that the timing of endotracheal intubation does not affect mortality and morbidity in critically ill COVID-19 patients [11]. Moreover, patients requiring endotracheal intubation after non-invasive mechanical ventilation (NIV) failure showed the same mortality rate compared to those intubated without NIV trial attempts [12].

Therefore, these results could justify a cautious “wait-and-see” approach, favoring the use of NIV as the primary respiratory support modality in critical COVID-19 patients. However, a scoping review performed by Radovanovic et al. [9] showed that few studies examined the role of NIV in critical COVID-19 patients requiring ICU admission. Furthermore, data on mortality in NIV patients admitted to ICUs are poorly reported, such as studies on clinical and laboratory predictors related to NIV failure or success in patients with COVID-19.

The aim of the present retrospective study is to analyze clinical and laboratory features present at ICU admission and able to predict NIV failure in COVID-19 patients.

## Material and method

### Patients’ enrollment

This retrospective observational study enrolled all patients admitted to a university hospital COVID ICU (“Da Procida” Hospital, Salerno, Italy) from 14th October 2020 to 30th April 2021. Inclusion criteria were age > 18 years old, positive reverse-transcriptase polymerase chain reaction nasopharyngeal swab test for SARS-CoV-2 infection, acute respiratory failure (defined as PaO_2_/FiO_2_ ≤ 300 [13]) eligible for NIV, ICU length of stay (LOS) ≥ 48 h. Exclusion criteria were ICU LOS < 48 h and other major causes requiring ICU admission, i.e., spontaneous cerebral hemorrhage, trauma, post-operative complications in COVID patients.

### NIV management and sedation protocol

A “patient eligible for NIV” is defined as a conscious and cooperative subject requiring NIV support by oral-nasal mask, full-face mask or helmet, in both pressure support ventilation (PSV) and continuous positive airway pressure (CPAP) modalities and not requiring endotracheal intubation within 3 h ICU admission. NIV was provided by conventional ICU mechanical ventilators. To enhance patients' compliance, a continuous infusion of dexmedetomidine (0.6–1.2 mcg/kg/h) and morphine bolus (5 mg iv) when required, was used. Sedation was adjusted stepwise to achieve a Richmond Agitation Sedation Scale (RASS) level of 0, -1 [14]. Awakening prone position ventilation was adopted according to patients' tolerance and aimed to be continued for 3 h at least [15].

**Fig. 1:**
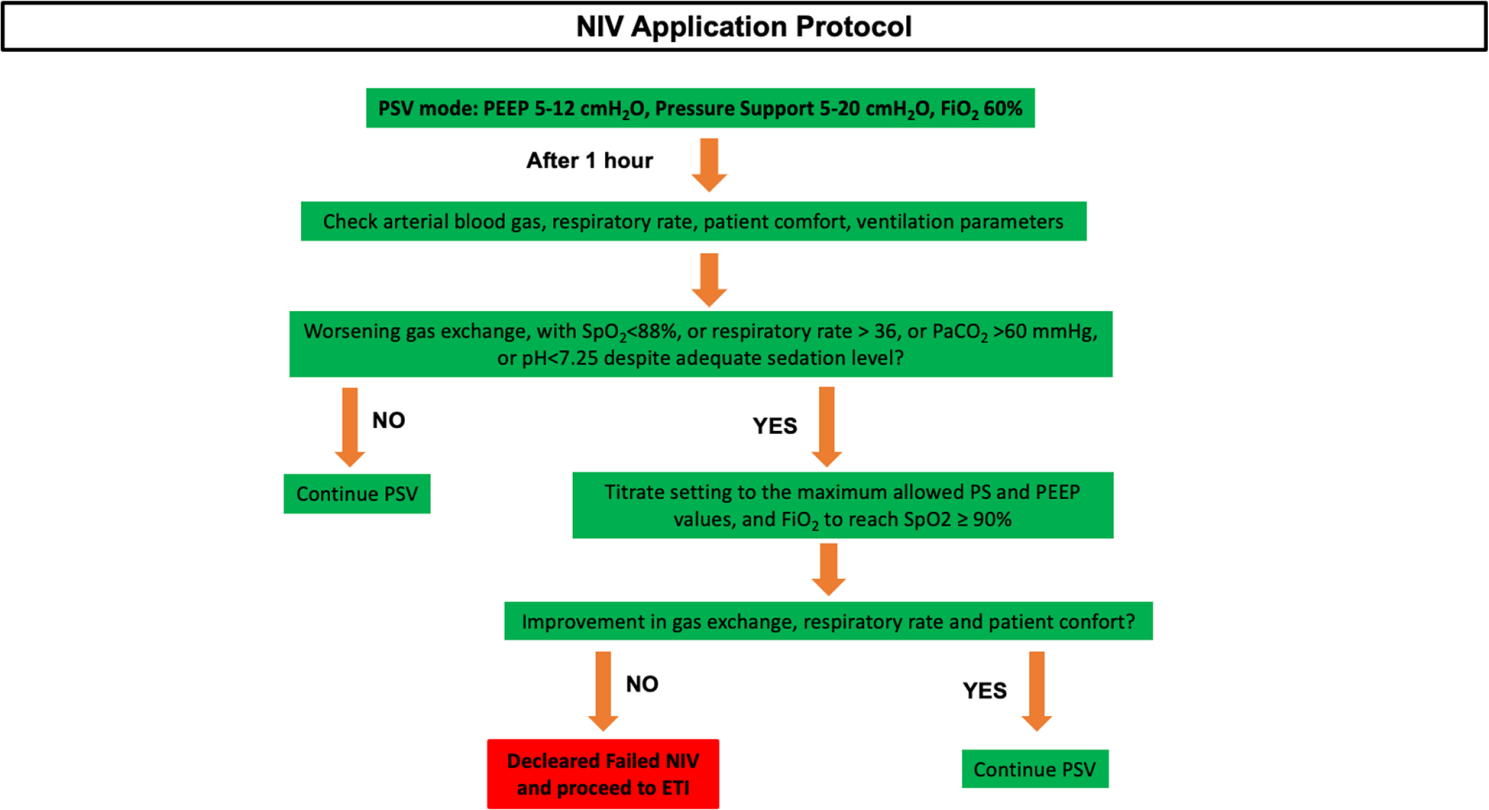
NIV protocol. The figure shows the NIV protocol adopted for the patients admitted to ICU. When patients started with CPAP mode at ICU admission, we set a PEEP value ranging between 5 and 12 cmH_2_O and FiO_2_ 60%. After 1 h, the clinician evaluated gas exchanges, by arterial blood gas analysis, respiratory rate, and patient’s comfort. In case of worsening gas exchange with SpO_2_ < 88%, or respiratory rate > 36, or PaCO_2_ > 60 mmHg, or pH < 7.25 despite adequate sedation level, the patient stopped CPAP and started NIV in PSV mode according to our protocol. On the contrary case, the patient continued CPAP mode ventilation

Figure 1 shows details about the NIV application protocol. NIV was continued whenever possible and based on the patient’s tolerance. When FiO_2_ was < 50%, respiratory rate < 30 breaths per minute, expiratory tidal volume > 5 mL/kg body weight expected with a pressure support < 10 cmH_2_O, and PEEP < 8 cmH_2_O, NIV was progressively suspended, and a high-flow nasal oxygen (HFNO) was started based on arterial blood gas (ABG) data. HFNO was also used during patients’ mobilization on a chair and meals [16].

NIV failure was defined according to ERS/ATS guidelines [17], as the persistence of a low PaO_2_/FiO_2_ ratio (less than 100 mmHg despite optimal NIV settings) and high respiratory rate (> 36/min). In order to avoid endotracheal intubation, NIV was titrated with a maximum pressure support of 20 cmH_2_O, maximum PEEP of 10–12 cmH_2_O and FiO_2_ set to obtain an oxygen saturation higher than 90%. We checked in the single patient in order to avoid barotrauma and intolerance.

In case of persistent or worsening of gas exchanges (oxygen saturation < 88%, respiratory rate > 36/min), patient’ inability to protect airways (i.e., coma or convulsive disorder) or to manage abundant tracheal and/or bronchial secretions, and hemodynamic or electrocardiographic instability, intensivists proceeded to endotracheal intubation and started invasive mechanical ventilation.

### Therapy

We provided all therapies as part of our standard care pathway. All patients, following the available scientific evidence, received: intravenous dexamethasone (6 mg iv, once a day for 10 days) and subcutaneous enoxaparin (50 I.U./kg, once a day as prophylaxis dosing, or bid in case of thrombosis suspected or confirmed). In addition, in selected cases and according to the best available evidence, we administered tocilizumab, remdesivir, and eculizumab.

### Data collection

Demographic, clinical, laboratory, imaging, and outcome data were extracted from electronic and paper medical records and anonymously collected on a digital sheet (Excel, Microsoft). A detailed description of collected data is provided in Supplementary File 1.

We used the Charlson Comorbidity Index (Supplementary File 2) to identify the chronic conditions which might impact long-term survival [18]. Sequential Organ Failure Assessment (SOFA) score [19] and neutrophil/lymphocyte ratio were calculated [20].

We reported the patient’s length of stay (LOS) in the pneumology/infectious disease ward, defined as the days from emergency room (ER) to ICU admission, ICU LOS, NIV-days, and ICU survival rates.

### Statistical analysis

We performed statistical analysis with MedCalc® Statistical Software version 19.6 (MedCalc Software Ltd, Ostend, Belgium; https://www.medcalc.org; 2020) and R software (R Core Team 2020, R: A language and environment for statistical computing. R Foundation for Statistical Computing, Vienna, Austria. URL https://www.R-project.org/).

No statistical sample size assessment was performed a priori, and the sample size was the number of patients requiring NIV during the study period, meeting inclusion and exclusion criteria. We reported the number of missing data and carried out the statistics based on available ones (Supplementary Table 1).

To investigate the influence of clinical and laboratory variables on the main outcome, NIV failure, we performed univariate Cox regression analysis. Categorical variables with a frequency ≤ 5 cases were excluded. First, Hazard-ratio (HR), 95% confidence interval (CI_95%_) and p-value were computed. Considering Charlson Comorbidity Index as an independent risk factor related to poor outcomes in COVID-19 patients [21], in a second step, each variable was adjusted for Charlson Comorbidity Index. Adjusted HR (HR_Adj_) with CI_95%_ were computed.

For significant models, the performance was analyzed with the receiver operating characteristics (ROC) curve [22]. The area under the curve (AUC) and CI_95%_ were computed. The best cut-off value using the Youden’s (*J*) index [23], sensibility (Se, defined as the true positive rate, and specificity (Sp, defined as the true negative rate), with CI_95%,_ were computed. Finally, we compared ROC curves for the single significative variable, adjusted variable for the Charlson Comorbidity Index, and Charlson Comorbidity Index. A *p*-value < 0.05 was considered statistically significant.

Furthermore, a subgroup analysis between patients who experienced NIV failure and those who did not was performed and details about statistical methods and results are provided in Supplementary file 1. We reported the data in tables and graphics.

## Results

On admission, 82 patients required NIV (70.1%), representing the studied sample. Table 1 reports the details of the population’s main characteristics.

**Table 1:** Demographic and clinical features of the sample

Main characteristics of the population (82 patients)	
**Demographic data**	
**Variable**	**Result**
*Sex, male (%)*	62 (75.6%)
*Age (years)*	67.0 [56.5–73.0]
*BMI (kg/m*^*2*^*)*	27.8 [25.3–31.3]
*NIV-day*	5.0 [3.0–8.5]
*Ward LOS (days)*	3.0 [0.0–7.0]
*ICU LOS (days)*	8.0 [5.0–12.0]
*Survived (%)*	37 (45.1%)
**Comorbidities**	
**Variable**	**Result**
*Charlson Comorbidity Index*	3 [1–4]
*Hypertension (%)*	45 (54.9%)
*Obesity (%)*	27 (32.9%)
*Diabets (%)*	22 (26.8%)
*COPD (%)*	15 (18.3%)
*CAD (%)*	10 (12.2%)
*CKD (%)*	4 (4.9%)
*Endocrinological disease (%)*	4 (4.9%)
*Atrial fibrillation (%)*	4 (4.9%)
*DVT (%)*	3 (3.7%)
*CVD (%)*	3 (3.7%)
*Asthma (%)*	3 (3.7%)
*Autoimmune disease (%)*	3 (3.7%)
*Hematological disease (%)*	2 (2.4%)
*OSAS (%)*	2 (2.4%)
*Neurological disorder (%)*	2 (2.4%)
*Liver disease (%)*	1 (1.2%)
**Arterial blood gas analysis and ventilation parameters**	
**Variable**	**Result**
*pH*	7.46 [7.43–7.48]
*PaO*_*2*_*(mmHg)*	89.5 [75.0–106.2]
*PaCO*_*2*_*(mmHg)*	37.5 [33.0–42.0]
*PaO*_*2*_*/FiO*_*2*_	125.0 [98.5–177.7]
*Lactate (mmol/L)*	1.3 [1.1–1.8]
*CPAP (%)*	13 (15.9%)
*PSV (%)*	69 (84.1%)
*PEEP (cmH*_*2*_*O)*	10 [10–10]
*Pressure support (cmH*_*2*_*O)*	7.5 [6.0–10.0]

The table reports demographic and clinical features. Frequencies are expressed as numbers and percentages (%). Continuous variables are expressed as mean ± standard deviation (SD) or median, first and third quartile [*q*_1_–*q*_3_]. *BMI*, body mass index; *LOS*, length of stay; *CAD*, coronary artery disease; *DVT*, deep vein thrombosis; *CVD*, cerebral-vascular disease; *CKD*, chronic kidney disease; *COPD*, chronic obstructive pulmonary disease; *OSAS*, obstructive sleep apnea syndrome; *CPAP*, continuous positive airway pressure; *PSV*, pressure support ventilation; *PEEP*, positive end-expiratory pressure

PSV was the most common modality used for NIV (84.1%), and the median NIV-day was 5.0 [3.0–8.5] (minimum and maximum, respectively, 1.0 and 18.0 days); 37 patients (45.1%) were discharged alive from the ICU. NIV-failed and NIV-successful groups consisted of 44 and 38 patients, respectively. Tables 2, 3, and 4 show detailed results of statistical comparison between the groups, such as described in Supplementary file 1. The survival rate in NIV-successful group resulted higher than NIV-failed group (94.7% vs 2.3%, *p*-value < 0.0001, Table 2).

**Table 2:** NIV-failed vs NIV-successful groups: demographic, comorbidities, blood gas analysis, and ventilation setting parameter statistical analysis

Variable	NIV-failed (***n*** = 44)	NIV-successful (***n*** = 38)	***p***-value
**Demographic data**			
*Male gender (%)*	28 (63.6%)	34 (89.5%)	*0.0069*
*Age (years)*	69.4 ± 7.8	59.0 ± 12.4	*< 0.0001*
*BMI (kg/m*^*2*^*)*	27.4 [25.1–31.0]	28.7 [25.2–38.0]	*0.1215*
*NIV day (days)*	5.0 [3.0–9.0]	4.5 [3.0–8.0]	*0.7931*
*Ward LOS (days)*	4.0 [0.0–7.0]	3.0 [0.0–7.0]	*0.8540*
*ICU LOS (day)*	10.0 [7.0–13.0]	5.0 [4.0–8.0]	*< 0.0001*
*Survived (%)*	1 (2.3%)	36 (94.7%)	*< 0.0001*
**Comorbidities**			
*Charlson Comorbidity Index*	4 [3–4]	2 [1–3]	*<0.0001*
*Hypertension (%)*	28 (63.6%)	17 (44.7%)	*0.0863*
*Obesity (%)*	12 (27.3%)	15 (39.5%)	*0.2411*
*Diabets (%)*	15 (34.1%)	7 (18.4%)	*0.1103*
*COPD (%)*	12 (27.3%)	3 (7.9%)	*0.0236*
*CAD (%)*	8 (18.2%)	2 (5.3%)	*0.0764*
*CKD (%)*	3 (6.8%)	1 (2.6%)	*0.3801*
*Endocrinological disease (%)*	4 (9.1%)	0 (0.0%)	*0.0567*
*Atrial fibrillation (%)*	3 (6.8%)	1 (2.6%)	*0.3801*
*DVT (%)*	2 (4.5%)	1 (2.6%)	*0.6453*
*CVD (%)*	3 (6.8%)	0 (0.0%)	*0.1010*
*Asthma (%)*	0 (0.0%)	3 (7.9%)	*0.0579*
*Autoimmune disease (%)*	3 (6.8%)	0 (0.0%)	*0.1010*
*Hematological disease (%)*	1 (2.3%)	1 (2.6%)	*0.9163*
*OSAS (%)*	1 (2.3%)	1 (2.6%)	*0.9163*
*Neurological disorder (%)*	2 (4.5%)	0 (0.0%)	*0.1833*
*Liver disease (%)*	1 (2.3%)	0 (0.0%)	*0.3498*
**Arterial blood gas analysis and ventilation parameters**			
*pH*	7.45 [7.44–7.49]	7.46 [7.43–7.48]	*0.8280*
*PaO*_*2*_*(mmHg)*	81.0 [74.0–106.7]	91.0 [77.2–104.4]	0.7977
*PaCO*_*2*_*(mmHg)*	36.0 [30.2–42.0]	39.0 [34.5–42.2]	0.2650
*Lactate (mmol/L)*	1.5 [1.3–1.9]	1.2 [1.0–1.6]	0.0070
*PaO*_*2*_*/FiO*_*2*_	111.0 [93.0–182.7]	127.0 [116.2–173.2]	0.2379
*CPAP (%)*	3 (6.8%)	10 (26.3%)	*0.0166*
*PEEP (cmH*_*2*_*O)*	10.0 [10.0–10.0]	10.0 [10.0–11.0]	*0.4785*
*Pressure support (cmH*_*2*_*O)*	6.0 [5.0–10.0]	9.0 [7.0–10.0]	*0.0393*

The table reports the statistical comparison of demographic, comorbidities, blood gas analysis, and ventilation setting parameters between the groups. Frequencies are expressed as numbers and percentages (%). Continuous variables are expressed as mean ± standard deviation (SD) or median, first and third quartile [*q*_1_–*q*_3_]. In case of missing data, statistics were performed on available data. Differences in frequencies were tested with the chi-square test. Differences in continuous variables were tested with two-tailed Student’s *t*-test (equal variance) or Welch’s test (unequal variance) or, for not normally distributed continuous variables, the Mann-Whitney test. All tests were performed with an *α* = 0.05, and a *p*-value < 0.05 was considered statistically significant

**Table 3:** NIV-failed vs NIV-successful groups: laboratory findings and therapies statistical analysis

Variable	Overall (***n*** = 82)	NIV-failed (***n*** = 44)	NIV-successful (***n*** = 38)	***p***-value
**Laboratory results**				
*Glicemia (mg/dL)*	139.0 [117.0–177.0]	160.5 [129.5–202.5]	123.5 [112.0–154.0]	*0.0039*
*Azotemia (mg/dL)*	60.5 [46.2–71.0]	63.5 [49.5–96.0]	60.0 [45.0–69.0]	*0.2284*
*Creatinine (mg/dL)*	0.74 [0.61–0.96]	0.73 [0.56–1.09]	0.74 [0.66–0.91]	*0.7802*
*eGFR (mL/min)*	95.5 [73.3–106.7]	91.5 [66.7–99.3]	100.8 [88.8–113.3]	*0.0104*
*Total protein (g/dL)*	6.0 ± 0.6	6.1 ± 0.6	6.1 ± 0.6	*0.7854*
*Albumin (g/dL)*	0.7 [0.6–0.9]	2.9 ± 0.3	3.1 ± 0.4	*0.0051*
*Bilirubin (mg/dL)*	3.0 ± 0.4	0.8 [0.6–0.9]	0.7 [0.5–0.9]	*0.4540*
*Ammonium (μg/dL)*	84.0 [65.0–113.0]	84.0 [63.0–110.7]	85.5 [67.5–115.5]	*0.9250*
*Sodium (mEq/L)*	138.0 [136.0–140.0]	138.0 [136.0–143.0]	138.0 [136.0–140.0]	*0.3287*
*Potassium (mEq/L)*	4.4 ± 0.5	4.2 ± 0.4	4.6 ± 0.5	*0.0013*
*Clorum (mEq/L)*	102.0 [99.5–104.0]	103.0 [100.2–105.7]	102.0 [99.0–103.0]	*0.1595*
*Calcium (mg/dL)*	8.5 [8.1–8.8]	8.4 [8.1–8.6]	8.6 [8.3–8.9]	*0.0443*
*Magnesium (mg/dL)*	2.2 ± 0.3	2.3 [2.0–2.5]	2.2 [2.0–2.4]	*0.8127*
*AST (U/L)*	31.5 [25.0–48.2]	29.0 [23.0–45.5]	34.0 [27.0–50.0]	*0.3218*
*ALT (U/L)*	37.0 [26.0–56.0]	30.0 [20.0–42.5]	49.5 [28.0–79.0]	*0.0016*
*LDH (U/L)*	413.0 [319.5–598.0]	456.5 [348.0–614.5]	387.5 [299.0–550.0]	*0.1344*
*CPK (U/L)*	69.5 [40.5–141.5]	61.5 [45.0–132.0]	82.0 [34.0–156.0]	*0.7168*
*Troponin (ng/L)*	11.3 [5.0–21.3]	13.5 [8.4–38.6]	6.0 [3.7–13.9]	*0.0008*
*Mioglobin (ng/mL)*	62.3 [37.8–110.9]	75.5 [39.2–169.5]	49.9 [32.5–91.5]	*0.0894*
*CK-MB (ng/mL)*	1.7 [1.1–2.8]	2.1 [1.3–3.9]	1.3 [0.9–1.9]	*0.0038*
*BNP (pg/mL)*	53.0 [33.0–123.2]	86.0 [46.5–196.2]	34.0 [21.0–58.0]	*0.0001*
*Hb (g/dL)*	13.7 [12.2–14.6]	13.1 [12.1–13.9]	14.3 [12.4–14.9]	*0.0327*
*WBC (× 10*^*3*^*)*	11.0 [8.1–13.3]	11.8 [9.1–13.3]	10.1 [7.6–13.3]	*0.1017*
*Neutrophil (× 10*^*3*^*)*	9.6 [7.0–11.7]	10.3 [8.0–12.0]	8.4 [6.9–10.9]	*0.0542*
*Lymphocytes*	727 [536–999]	758 ± 330	850 ± 348	*0.2255*
*Eosinophil*	85 [48–154]	96 [53–156]	78 [39–149]	*0.2906*
*Monocyte*	437 [318–627]	433 [317–580]	476 [322–700]	*0.5121*
*Basophil*	13 [8–24]	13 [10–24]	12 [5–21]	*0.2182*
*Neutrophil/lymphocyte ratio*	12.1 [8.8–18.0]	15.1 [10.4–19.7]	10.3 [7.4–14.4]	*0.0080*
*Platelet (× 10*^*3*^*)*	266.6 ± 103.9	247.4 ± 104.6	288.8 ± 99.9	*0.0714*
*aPTT (sec)*	29.9 [26.8–32.8]	31.2 [29.3–34.0]	27.9 [25.9–30.6]	*0.0041*
*INR*	1.13 [1.04–1.23]	1.19 [1.04–1.31]	1.12 [1.04–1.18]	*0.0921*
*Firbinogen (mg/dL)*	538.5 [471.0–670.0]	505.5 [424.5–588.0]	596.0 [512.0–699.0]	*0.0166*
*D-dimer (ng/mL)*	1060.0 [680.7–2495.0]	1460.0 [860.0–3825.0]	820.0 [530.0–1260.0]	*0.0037*
*AT III (%)*	86.3 ± 15.9	82.4 ± 16.2	90.8 ± 14.4	*0.0161*
*CRP (mg/dL)*	7.3 [3.6–12.7]	8.9 [5.1–14.2]	6.1 [3.6–9.4]	*0.0762*
*PCT (ng/mL)*	0.10 [0.06–0.23]	0.13 [0.09–0.35]	0.08 [0.05–0.14]	*0.0141*
*SOFA score*	3 [3–4]	4 [3–5]	3 [3–4]	0.0808
**Therapies**				
*Tocilizumab (%)*	16 (19.5%)	6 (13.6%)	10 (26.3%)	*0.1490*
*Eculizumab (%)*	2 (2.4%)	1 (2.3%)	1 (2.6%)	*0.9163*
*Remdesivir (%)*	6 (7.3%)	2 (4.5%)	4 (10.5%)	*0.2997*

The table reports the statistical comparison of laboratory findings and therapies between the groups. Frequencies are expressed as numbers and percentages (%). Continuous variables are expressed as mean ± standard deviation (SD) or median, first and third quartile [*q*_1_–*q*_3_]. In case of missing data, statistics were performed on available data. Differences in frequencies were tested with the chi-square test. Differences in continuous variables were tested with two-tailed Student’s *t*-test (equal variance) or Welch’s test (unequal variance) or, for not normally distributed continuous variables, the Mann-Whitney test. All tests were performed with an *α* = 0.05, and a *p*-value < 0.05 was considered statistically significant

**Table 4:** NIV-failed and NIV-successful groups: chest CT scan feature statistical analysis

Variable	Overall (***n*** = 58)	NIV-failed (***n*** = 31)	NIV-successful (***n*** = 27)	***p***-value
*≥ 4 involved lobes (%)*	54 (93.1%)	28 (90.3%)	26 (96.3%)	*0.3705*
*Ground-glass opacity (%)*	51 (87.9%)	30 (96.8%)	21 (77.8%)	*0.0281*
*Consolidation (%)*	43 (74.1%)	21 (67.7%)	22 (81.5%)	*0.2373*
*Lymphoadenopathy (%)*	27 (46.6%)	16 (51.6%)	11 (40.7%)	*0.4117*
*Interstitial septum thickening (%)*	12 (20.7%)	8 (25.8%)	4 (14.8%)	*0.3068*
*Crazy paving (%)*	8 (13.8%)	3 (9.7%)	5 (18.5%)	*0.3343*
*Pneumomediastinum (%)*	9 (15.5%)	7 (22.6%)	2 (7.4%)	*0.1145*
*Bronchogram (%)*	6 (10.3%)	4 (12.9%)	2 (7.4%)	*0.4968*
*Pleural effusion (%)*	8 (13.8%)	3 (9.7%)	5 (18.5%)	*0.3343*
*Emphysema (%)*	8 (13.8%)	6 (19.4%)	2 (7.4%)	*0.1920*
*Pneumothorax (%)*	5 (8.6%)	3 (9.7%)	2 (7.4%)	*0.7607*
*Cavitation (%)*	5 (8.6%)	3 (9.7%)	2 (7.4%)	*0.7607*
*Adjacent pleural thickening (%)*	7 (12.1%)	7 (22.6%)	0 (0.0%)	*0.0090*
*Subcutaneous emphysema (%)*	6 (10.3%)	6 (19.4%)	0 (0.0%)	*0.0167*
*Pericardial effusion (%)*	3 (5.2%)	1 (3.2%)	2 (7.4%)	*0.4771*
*Iodinate contrast (%)*	28 (48.3%)	17 (54.8%)	11 (40.7%)	*0.2880*
*Pulmonary thromboembolism (%)*	2 (7.1%)	2 (11.8%)	0 (0.0%)	*0.2378*

The table reports the statistical comparison of chest CT scan features between the groups. Frequencies are expressed as numbers and percentages (%). Differences in frequencies were tested with the chi-square test (*α* = 0.05) and a *p*-value < 0.05 was considered statistically significant

Single-variable Cox regression analysis showed that age, the Charlson Comorbidity Index, suffering from COPD and CAD, calcium, CPK, troponin, CK-MB, INR, and AT III were factors related to NIV failure (for details, see Table 5). When single variables were adjusted for the Charlson Comorbidity Index, only PaO_2_/FiO_2_ (HR_Adj_ 0.99, CI_95%_ 0.98–1.00, *p*-value 0.0181), CPK (HR_Adj_ 1.00, CI_95%_ 1.00–1.00, *p*-value 0.0064), INR (HR_Adj_ 2.32, CI_95%_ 1.10–4.85, *p*-value 0.0262), and AT III (HR_Adj_ 0.98, CI_95%_ 0.96–0.99, *p*-value 0.0249) showed a significant relationship with the considered outcome.

**Table 5:** Cox regression analysis

Variable	Univariate Cox regression		Multivariate Cox regression (adjusted for the Charlson Comorbidity Index)	
	HR (CI_**95%**_)	***p***-value	HR_**Adj**_ (CI_**95%**_)	***p***-value
*Gender, male*	0.82 (0.42–1.59)	*0.5533*	0.84 (0.43–1.65)	*0.6143*
*Age*	1.04 (1.00–1.07)	*0.0372*	1.00 (0.95–1.05)	*0.9315*
*BMI*	0.99 (0.92–1.05)	*0.6700*	1.00 (0.94–1.07)	*0.9212*
*Ward LOS*	1.00 (0.97–1.03)	*0.9638*	0.99 (0.96–1.03)	*0.7481*
*Charlson Comorbidity Index*	1.32 (1.10–1.57)	*0.0024*	–	*–*
*Hypertension*	1.11 (0.58–2.12)	*0.7499*	0.81 (0.41–1.59)	*0.5380*
*Obesity*	1.34 (0.65–2.78)	*0.4287*	1.76 (0.83–3.74)	*0.1381*
*Diabets*	1.70 (0.89–3.26)	*0.1065*	0.98 (0.45–2.13)	*0.9592*
*COPD*	2.33 (1.13–4.79)	*0.0212*	1.51 (0.69–3.30)	*0.2993*
*CAD*	2.56 (1.16–5.64)	*0.0199*	1.81 (0.80–4.11)	*0.1535*
*pH*	0.15 (0.00–33.93)	*0.4953*	17.11 (0.06–4463.02)	*0.3170*
*PaO*_*2*_	1.00 (0.99–1.01)	*0.7803*	0.99 (0.98–1.00)	*0.0843*
*PaCO*_*2*_	0.98 (0.94–1.03)	*0.4053*	1.00 (0.96–1.03)	*0.9155*
*Lactate*	1.36 (0.83–2.24)	*0.2244*	0.88 (0.50–1.57)	*0.6719*
*PaO*_*2*_*/FiO*_*2*_	1.00 (0.99–1.00)	*0.3118*	0.99 (0.98–1.00)	*0.0181*
*FiO*_*2*_	4.22 (0.58–30.40)	*0.1531*	5.52 (0.66–46.00)	*0.1146*
*Ventilation mode, PSV*	2.43 (0.74–7.92)	*0.1410*	2.07 (0.63–6.84)	*0.2332*
*PEEP*	1.10 (0.86–1.40)	*0.4339*	1.11 (0.85–1.46)	*0.4305*
*Pressure support*	0.89 (0.77–1.02)	*0.0852*	0.91 (0.79–1.04)	*0.1846*
*Glicemia*	1.00 (0.99–1.01)	*0.0714*	1.00 (1.00–1.00)	*0.8372*
*Azotemia*	1.00 (0.99–1.01)	*0.7315*	0.99 (0.99–1.00)	*0.3478*
*Creatinine*	1.09 (0.63–1.88)	*07611*	0.69 (0.39–1.23)	*0.2094*
*eGFR*	0.99 (0.98–1.01)	*0.4092*	1.00 (0.99–1.02)	*0.3930*
*Total protein*	1.28 (0.52–3.16)	*0.5907*	1.13 (0.47–2.71)	*0.7744*
*Albumin*	0.49 (0.21–1.17)	*0.1079*	0.52 (0.20–1.32)	*0.1673*
*Bilirubin*	1.08 (0.80–1.45)	*0.6109*	0.99 (0.74–1.32)	*0.9494*
*Ammonium*	1.00 (0.99–1.01)	*0.9765*	1.00 (0.99–1.01)	*0.6060*
*Sodium*	1.00 (0.96–1.05)	*0.8205*	1.00 (0.95–1.04)	*0.9191*
*Potassium*	0.60 (0.33–1.08)	*0.0883*	0.75 (0.41–1.37)	*0.3536*
*Clorum*	1.00 (0.93–1.06)	*0.9048*	0.98 (0.92–1.05)	*0.6350*
*Calcium*	0.51 (0.28–0.93)	*0.0288*	0.55 (0.29–1.03)	*0.0626*
*Magnesium*	0.68 (0.27–1.71)	*0.4110*	0.92 (0.36–2.34)	*0.8621*
*AST*	1.01 (0.99–1.02)	*0.4030*	1.01 (0.99–1.02)	*0.2631*
*ALT*	0.99 (0.98–1.00)	*0.0599*	0.99 (0.98–1.00)	*0.1900*
*LDH*	1.00 (1.00–1.00)	*0.3968*	1.00 (1.00–1.00)	*0.4288*
*CPK*	1.00 (1.00–1.00)	*0.0491*	1.00 (1.00–1.00)	*0.0064*
*Troponin*	1.00 (1.00–1.00)	*0.0323*	1.00 (0.99–1.00)	*0.0823*
*Mioglobin*	1.00 (1.00–1.00)	*0.4230*	1.00 (1.00–1.00)	*0.9568*
*CK-MB*	1.08 (1.01–1.16)	*0.0269*	1.06 (0.98–1.15)	*0.1271*
*BNP*	1.00 (1.00–1.00)	*0.1776*	1.00 (1.00–1.00)	*0.7786*
*Hb*	0.86 (0.69–1.07)	*0.1800*	0.95 (0.75–1.20)	*0.6466*
*WBC*	1.00 (1.00–1.00)	*0.7677*	1.00 (1.00–1.00)	*0.6782*
*Neutrophil*	1.00 (1.00–1.00)	*0.7523*	1.00 (1.00–1.00)	*0.7071*
*Limphocyte*	1.00 (1.00–1.00)	*0.3318*	1.00 (1.00–1.00)	*0.2168*
*Eosinophil*	1.00 (1.00–1.00)	*0.4216*	1.00 (1.00–1.00)	*0.7185*
*Monocyte*	1.00 (1.00–1.00)	*0.7885*	1.00 (1.00–1.00)	*0.7783*
*Basophil*	1.00 (1.00–1.01)	*0.2188*	1.00 (0.99–1.00)	*0.4400*
*Neutrophil/lymphocyte ratio*	1.02 (0.99–1.06)	*0.1794*	1.02 (0.99–1.06)	*0.2176*
*Platelet*	1.00 (1.00–1.00)	*0.2903*	1.00 (1.00–1.00)	*0.8352*
*aPTT*	1.03 (0.99–1.06)	*0.1466*	1.03 (0.99–1.08)	*0.1072*
*INR*	2.60 (1.27–5.34)	*0.0090*	2.32 (1.10–4.85)	*0.0262*
*Firbinogen*	1.00 (1.00–1.00)	*0.3873*	1.00 (1.00–1.00)	*0.8126*
*D-dimer*	1.00 (1.00–1.00)	*0.1273*	1.00 (1.00–1.00)	*0.5689*
*AT III*	0.97 (0.95–0.99)	*0.0015*	0.98 (0.96–0.99)	*0.0249*
*CRP*	1.00 (1.00–1.01)	*0.8349*	1.00 (1.00–1.01)	*0.9794*
*PCT*	1.05 (0.99–1.11)	*0.0758*	1.06 (0.99–1.12)	*0.0738*
*SOFA*	1.21 (0.91–1.61)	*0.1807*	1.08 (0.83–1.40)	*0.5789*
*Tocilizumab*	0.57 (0.24–1.36)	*0.2053*	0.72 (0.30–1.76)	*0.4737*
*Remdesivir*	0.73 (0.18–3.05)	*0.6699*	0.96 (0.23–4.08)	*0.9607*

The table reports the results of univariate and multivariate (considering the Charlson Comorbidity Index) Cox regression analysis for single variables. Hazard ratio (HR), adjusted HR (HR_Adj_) with CI_95%_ were computed. A *p*-value < 0.05 was considered statistically significant

**Fig. 2:**
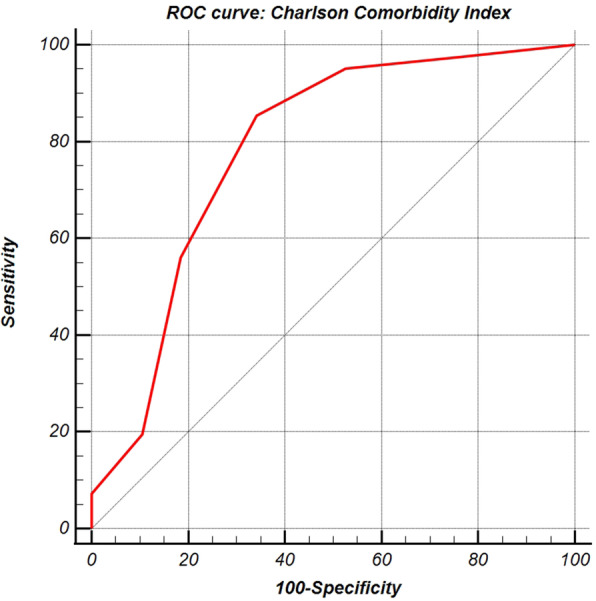
ROC curve analysis for the Charlson Comorbidity Index. The figure shows ROC curve analysis for the Charlson Comorbidity Index (AUC 0.784, CI_95%_ 0.677–0.869, *p*-value < 0.0001). The coefficient for the score and cut-off values were: score model [Charlson] = 0.2762 * (Charlson), cut-off values > 0.5523 (*J*-index = 0.5116). Se and Sp were, respectively 85.4% (CI_95%_ 70.8–94.4%) and 65.8% (CI_95%_ 48.6–80.4%)

**Fig. 3:**
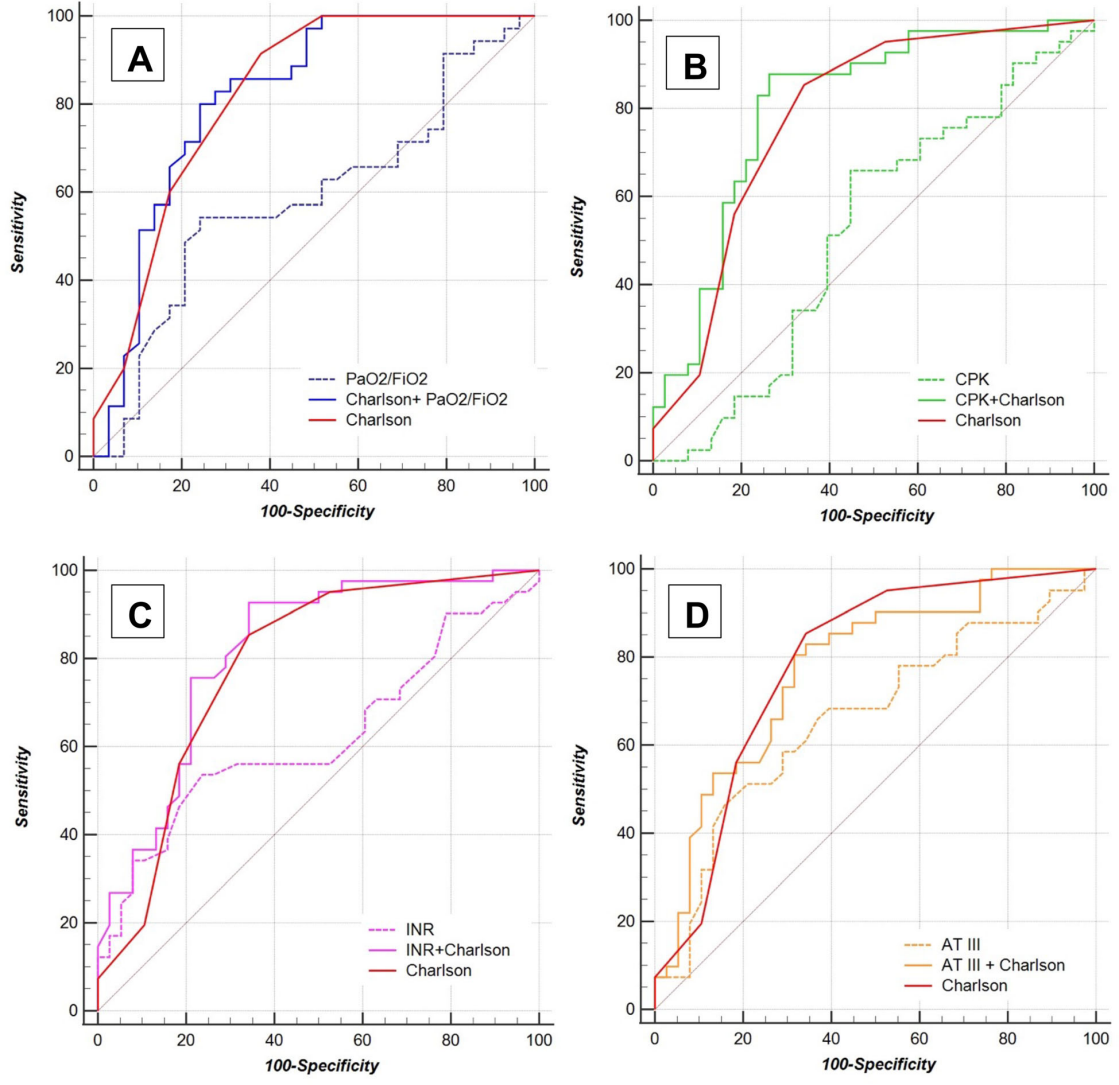
ROC curves analysis for single variables and adjusted for the Charlson Comorbidity Index. The figure shows ROC curve analysis for PaO_2_/FiO_2_ (**A**), for CPK (**B**), INR (**C**), and AT III (**D**). **A** Score model [PaO_2_/FiO_2_] = − 0.0028 * (PaO_2_/FiO_2_), cut-off values > − 0.3271 (*J*-index = 0.3015), Se 54.9% (CI_95%_ 36.6–71.2%) and Sp 75.9% (CI_95%_ 56.5–89.7%); score model [Charlson + PaO_2_/FiO_2_] = 0.4705 * (Charlson) − 0.0072 * (PaO_2_/FiO_2_), cut-off values > 0.2659 (*J*-index = 0.5586), Se 80.0% (CI_95%_ 63.1–91.6%) and Sp 75.9% (CI_95%_ 56.5–89.7%). The AUCs for single and adjusted model were, respectively, 0.586 (CI_95%_ 0.456-0.708, *p*-value 0.2400) and 0.819 (CI_95%_ 0.702–0.904, *p*-value < 0.0001). The adjustment for the Charlson Comorbidity Index showed a statistically significant difference for the AUC (*p*-value < 0.0001). When the Charlson Comorbidity Index and adjusted model score ROC curves were compared, the *p*-value was not statistically significant (0.9254). **B** Score model [CPK] = 0.0007 * (CPK), cut-off values ≤ 0.0558 (*J*-index = 0.2112), Se 65.8% (CI_95%_ 49.4–79.9%), Sp 55.3% (CI_95%_ 38.3–71.4%); score model [Charlson + CPK] = 0.2984 * (Charlson) + 0.0009 * (CPK), cut-off value > 0.9267 (*J*-index = 0.6149), Se 87.8% (CI_95%_ 73.8–95.9%), Sp 73.7% (CI_95%_ 56.9–86.6%). The AUCs for single and adjusted models were, respectively, 0.522 (CI_95%_ 0.407–0.636, *p*-value 0.7390) and 0.807 (CI_95%_ 0.703–0.887, *p*-value < 0.0001). The adjustment for Charlson Comorbidity Index showed a statistically significant difference for the AUC (*p*-value 0.0008). When the Charlson Comorbidity Index and adjusted model scores ROC curves were compared, the *p*-value was not statistically significant (*0.3234*). **C** Score model [INR] = 0.9573 * (INR), cut-off values > 1.1296 (*J*-index = 0.2997) Se 53.7% (CI_95%_ 37.4–69.3%), Sp 76.3% (CI_95%_ 59.8–88.6%); score model [Charlson +INR] = 0.2623 * (Charlson) + 0.8398 * (INR), cut-off value > 1.5220 (*J*-index = 0.5847), Se 92.7% (CI_95%_ 80.1–98.5%), Sp 65.8% (CI_95%_ 48.6–80.4%). The AUCs for single and adjusted model were, respectively, 0.621 (CI_95%_ 0.505–0.728, *p*-value 0.0590) and 0.815 (CI_95%_ 0.712–0.894, *p*-value < 0.0001). The adjustment for Charlson Comorbidity Index showed a statistically significant difference for the AUC (*p*-value 0.0051). When the Charlson Comorbidity Index and adjusted model scores ROC curves were compared, the *p*-value was not statistically significant (0.0805). **D** Score model [AT III] = − 0.0299 * (AT III), cut-off values > − 2.3655 (*J*-index = 0.3055), Se 46.3% (CI_95%_ 30.7–62.6%), Sp 84.2% (CI_95%_ 68.7–94.0%); score model [Carlson + AT III] = 0.1980 * (Charlson) − 0.0227 * (AT III), cut-off value > − 1.5922 (*J*-index = 0.4891), Se 80.5% (CI_95%_ 65.1–91.2%), Sp 68.4% (CI_95%_ 51.3–82.5%). The AUCs for single and adjusted models were, respectively, 0.662 (CI_95%_ 0.547–0.765, *p*-value 0.0092) and 0.776 (CI_95%_ 0.668–0.862, *p*-value < 0.0001). The adjustment for the Charlson Comorbidity Index showed a statistically significant difference for the AUC (*p*-value 0.0053). When the Charlson Comorbidity Index and adjusted model scores ROC curves were compared, the *p*-value was not statistically significant (0.8428)

Briefly, Charlson Comorbidity Index ROC curve analysis (Fig. 2) showed an AUC of 0.784 (CI_95%_ 0.677–0.869, *p*-value < 0.0001), with a Se and Sp for cut-off value of, respectively 85.4% (CI_95%_ 70.8–94.4%) and 65.8% (CI_95%_ 48.6–80.4%). For all significant variables, the adjustment for Charlson Comorbidity Index showed an increase in AUC statistically significant (see Fig. 3). Charlson Comorbidity Index + AT III model (Fig. 3D) showed an AUC of 0.776 (CI_95%_ 0.668–0.862, *p*-value < 0.0001), with a Se and Sp for cut-off value of, respectively, 80.5% (CI_95%_65.1–91.2%) and 68.4% (CI_95%_51.3–82.5%). However, even single AT III model score showed a significant statistically AUC (0.662, CI_95%_ 0.547–0.765, *p*-value 0.0092), with a Se 46.3% (CI_95%_ 30.7–62.6%) and Sp 84.2% (CI_95%_ 68.7–94.0%) for the cut-off value.

## Discussion

Several studies have been published on COVID-19 patients who underwent NIV outside ICU [9], while data are still required about NIV performed in the ICU setting. The NIV failure rate in ICU ranged from 17 to 47% [24, 25], with a mortality rate from 14 to 97% [12, 25, 26]. In this retrospective study, we showed that NIV was used in a large proportion of patients (70.1%) admitted to ICU to treat acute respiratory failure due to COVID-19, with a failure rate of 53.7%. Furthermore, patients who experienced failed NIV showed a higher mortality rate. Our data is in agreement with the evidence present in the international literature [12, 24–26].

Remarkably, patients who experienced NIV failure did not show a shorter NIV duration than patients who did not. Therefore, the correct patient selection based on clinical, laboratory, and imaging features can represent the “cornerstone” to reduce at least ICU distress, avoiding extenuating NIV “trial.” Our analysis points to suggest that patients who failed NIV represented a particular cluster, showing peculiar characteristics already presented at ICU admission that can be easily identified.

Patients who experienced NIV failure showed a higher Charlson Comorbidity Index than patients who did not. This data suggested that age and previous comorbidities, in detail, COPD, reduced patients’ reserve to respond to COVID-19-related inflammatory state. Furthermore, as showed by Cox regression analysis, Charlson Comorbidity Index represented a robust predictive factor related to NIV failure, with a high sensibility for cut-off value. The Charlson Comorbidity Index originally was developed to predict the risk of mortality within one year of hospitalization. During the current pandemia, the Charlson Comorbidity Index score, which considers the effects of both age and comorbidity, predicts death among COVID-19 patients by an exponential increase in the odds ratio at each score point [21]. Thus, the application of Charlson Comorbidity Index scoring in the context of the COVID-19 outbreak can be helpful to predict which ICU patient will experience NIV failure.

In our analysis, patients who experienced NIV failure showed the features of ongoing multiorgan impairment, expression of systemic disease, not only related to lung site. As reported by Zannella et al. [27], early multiorgan impairment due to the COVID-19 disease was already present at ICU admission, and it subsequently worsened during the ICU stay, mainly in non-survivors. In univariate Cox regression analysis, organ-specific injury markers, in detail cardiac and coagulation parameters, were able to predict NIV failure. However, when univariate models were adjusted for the Charlson Comorbidity Index, only PaO_2_/FiO_2_, CPK, INR, and AT III were single parameters able to predict NIV failure. Adjusted predictive models presented a good performance, as showed by AUC, with high sensibility and variable specificity for the cut-off values. When single variables were adjusted for the Charlson Comorbidity Index, the increase in AUC resulted statistically significant, but the comparisons between adjusted and single Charlson Comorbidity Index model scores did not show a statistically significant difference.

In our study PaO_2_/FiO_2_value at ICU admission did not show a statistically significant difference between the two groups but, instead, in the univariate Cox regression analysis, it resulted significantly related to NIV failure when adjusted for Charlson Comorbidity Index. Grasselli et al. [28], in a retrospective study, showed that PaO_2_/FiO_2_ was higher in younger patients than older patients, with an increased mortality rate in the latter. Chen et al. [29] reported that initial PaO_2_/FiO_2_< 122.17 mmHg should be considered a “warning sign” in patients with COVID-19 and guide the clinician to evaluate the need for endotracheal intubation and invasive mechanical ventilation. Coppadoro et al. [30] demonstrated that PaO_2_/FiO_2_ collected during helmet CPAP treatment and the number of comorbidities was independently associated with NIV failure. According to our results, PaO_2_/FiO_2_ should not be used as a single parameter to predict NIV failure but should be implemented with Charlson Comorbidity Index. The influence of the Charlson Comorbidity Index on the PaO_2_/FiO_2_ is poorly studied, and further analyses are needed.

In addition to lung damage, muscle weakness and elevation of serum CPK level were documented in around 20% severe SARS-CoV2 infection and could be interpreted as a manifestation of multiorgan damage [31]. In the present study, CPK levels between groups did not show a statistically significant difference. Still, both single and bivariate, Cox regression analysis showed that CPK levels could be considered a parameter able to predict NIV failure. Even though several factors could cause an increase in serum CPK, the pathogenesis of muscle damage in such patients remains unknown. It was suggested that a possible myotoxic effect of SARS-CoV2 should be carefully assessed particularly in severe SARS-CoV2 infection [32]. Our data indicated that serum CPK increasing could be considered a potential prognostic sign for muscle weakness and, consequently, failed NIV. Further investigations are necessary regarding this topic.

Evidence suggests that SARS-CoV-2 can cause a series of acquired coagulation disorders, producing endothelial damage, coagulation activation, and intravascular fibrin deposition. For severe COVID-19 patients, coagulation activation can lead to thrombus formation and even disseminated intravascular coagulation [33]. In our study, when adjusted for the Charlson Comorbidity Index, INR and AT III level were related to NIV failure, suggesting that early coagulation impairment and subliminal parameters alterations should be considered markers of severity or an ongoing stadium of the disease. It is interesting to note that D-dimer, the hallmark of severe COVID-19 patients, was not related to failed NIV, suggesting that alteration in INR and AT III could anticipate the “catastrophic” increase in D-dimer levels. Our results are in line with those reported in previous studies. Ouyang et al. [34], analyzing the temporal changes in laboratory markers of adult COVID-19 survivors and non-survivors, reported that INR was higher in non-survivors both in the first and latter tests. In the present analysis, AT III levels resulted higher in patients who had successful NIV and the single score model showed an AUC statistically significant, with a high specificity. Our results are in line with Tang et al. [35], reporting a statistically significant reduction in AT III occurred in non-survivors of COVID-19 patients compared to survivors after day 7 of admission. This reduction seems to persist until day 14.

Although we did not include chest CT findings in our model, ground glass opacity, adjacent pleural thickening, and subcutaneous emphysema presented a higher incidence in patients who experienced NIV failure. Ground glass opacity represents the most common CT imaging feature in patients with COVID-19 pneumonia [36]. At the same time, subcutaneous emphysema should be considered a premonitory sign for the development of more severe barotrauma. Subcutaneous emphysema should be encompassed in a range of clinical manifestations defined as “alveolar air leaks syndrome” [37]. Further findings are essential to clarify if its occurrence represents a severe index disease or the results of an inappropriate NIV setting [38].

## Conclusions

Although NIV was extensively used in ICU as respiratory support to treat COVID-19 related ARDS, its failure is associated with high mortality. To stratify patients, it can be helpful to have predictive factors, available at the ICU admission. In the present analysis, factors able to identify patients at risk for NIV failure at ICU admission were the Charlson Comorbidity Index and its combination with PaO_2_/FiO_2_, CPK, INR, and AT III. Derived models showed higher AUC and higher sensibility for the cut-off point, suggesting a potential role for the identification of patients considered at risk of NIV failure. The main limitation of this study is represented by its retrospective design and its small sample size. Moreover, we provided a description of our population at ICU admission, and we did not evaluate how variables changed during ICU stay. Further studies are needed to clarify the role of endotracheal intubation and invasive mechanical ventilation to reduce mortality in the population considered at risk of NIV failure [39].

## Supplementary Information

**Additional file 1: Supplementary file 1.**

**Additional file 2: Supplementary file 2.**

**Additional file 3: Supplementary Table 1.**

## Data Availability

The datasets used and analyzed during the current study are available from the corresponding author on reasonable request.

## Abbreviations

CI_95%_: 95% confidence interval
aPTT: Activated partial thromboplastin time
ARDS: Acute respiratory distress syndrome
HR_Adj_: Adjusted HR
AST: Alanine-amine transferase
AT III: Antithrombin III
AUC: Area under the curve
ALT: Aspartate-amine transferase
BMI: Body mass index
BNP: Brain natriuretic peptide
Ca: Calcium
CT: Chest-tomography
*Χ*^2^: Chi-square test
CKD: Chronic kidney disease
COPD: Chronic obstructive pulmonary disease
Cl: Clorum
PaCO_2_: CO_2_partial pressure
CPAP: Continuous positive airway pressure
CAD: Coronary artery disease
COVID-19: Coronavirus disease 2019
CPK: Creatine kinase
eGFR: Estimated glomerular filtration rate
Hb: Hemoglobin concentration
HR: Hazard-ratio
INR: International normalized ratio
LDH: Lactate dehydrogenase
LOS: Length of stay
Mg: Magnesium
max: Maximum
CK-MB: MB creatine kinase isoform
min: Minimum
NIV: Non-invasive mechanical ventilation
FiO_2_: O_2_ fraction inspired
PaO_2_: O_2_ partial pressure
OSAS: Obstructive sleep apnea syndrome
PLT: Platelets
PEEP: Positive end-expiratory pressure
K: Potassium
PSV: Pressure support ventilation
ROC: Receiver operating characteristics
SILI: Self-inflicted lung injury
Se: Sensibility
SOFA: Sequential Organ Failure Assessment
SARS-CoV-2: Severe acute respiratory syndrome coronavirus 2
Na: Sodium
Sp: Specificity
SD: Standard deviation
TnI: Troponin I
WBC: White blood cell count
WHO: World Health Organization
*J*: Youden’s index
