# Supplementary material for: Predictor factors for non-invasive mechanical ventilation failure in severe COVID-19 patients in the intensive care unit: a single-center retrospective study
Source: J Anesth Analg Crit Care. 2022 Feb 15;2:10. doi: 10.1186/s44158-022-00038-7 (PMC8853166; doi:10.1186/s44158-022-00038-7)
Supplement: Supplementary file 1 — Additional file 1: Supplementary file 1. [file 44158_2022_38_MOESM1_ESM.docx]

**Supplementary file 1**

**Data Collection**

Sex (male/female), age, and body mass index (BMI, calculated as weight, in kilograms, divided by the square of the height, in meters) were collected. We used the Charlson Comorbidity Index to identify the chronic conditions which might impact long-term survival [1]. We reported the following coexisting conditions: chronic obstructive pulmonary disease (COPD), restrictive or interstitial pulmonary disease; history of hypertension or cardiovascular disease, defined as coronary artery disease (CAD), heart failure, valvular disease, and cardiac arrhythmia; obesity (defined as BMI ≥ 30 kg/m^2^), diabetes, endocrinological disease, chronic kidney disease (CKD), liver disease (viral or autoimmune hepatitis), neurological disorders (epilepsy, Parkinson’s disease or dementia), autoimmune disease (i.e. arthritis rheumatoid), haematological malignancy (lymphoma or leukaemia), solid neoplasia, solid-organ transplantation, previous deep vein thrombosis and obstructive sleep apnea syndrome (OSAS).

The O_2_ partial pressure (PaO_2_), CO_2_ partial pressure (PaCO_2_), O_2_ fraction inspired (FiO_2_), lactate, arterial pH, and PaO_2_/FiO_2_, and NIV modality, PEEP and pressure support values were reported.

Blood samples were performed at ICU admission, and the following data were collected:

- Blood glucose, creatinine, azotemia, estimated glomerular filtration rate (eGFR, calculated by Cockcroft-Gault formula), sodium (Na), potassium (K), clorum (Cl), calcium (Ca), and magnesium (Mg);
- Troponin I (TnI), brain natriuretic peptide (BNP), myoglobin, creatine kinase (CPK), and MB creatine kinase isoform (CK-MB);
- Alanine-amine transferase (AST), aspartate-amine transferase (ALT), lactate dehydrogenase (LDH), total bilirubin, protein, albumin, and blood ammonium;
- Cell Blood Count: white blood cell count (WBC) and leukocyte formula (absolute number of neutrophils, lymphocytes, eosinophils, basophils, and monocytes), haemoglobin concentration (Hb), platelets (PLT);
- Coagulation parameters: activated partial thromboplastin time (aPTT), international normalized ratio (INR), D-dimer, fibrinogen, antithrombin III (AT III);
- Inflammatory marker: C Reactive Protein (CRP) and procalcitonin (PCT).

Sequential Organ Failure Assessment (SOFA) score[2] and Neutrophil/Lymphocyte Ratio were calculated [3].

We reported a qualitative description of chest-tomography (CT) scan performed within four days after ICU admission or, if not available, up to four days before ICU admission. Qualitative analysis of CT scan consisted in reporting the following items described by a radiologist [4]:

- Number of involved pulmonary lobes;
- Lesions pattern: ground-glass opacity, consolidation, crazy paving pattern, cavitation;
- Pleural and pericardial effusion, adjacent pleura thickening, lymphadenopathy, pulmonary emphysema, and, when iodinate contrast was used, pulmonary embolism;
- Barotrauma signs: pneumomediastinum, pneumopericardium, pneumothorax, subcutaneous emphysema;
- Other signs: interlobular septal thickening, combined linear opacities, bronchogram.

**Subgroup statistical analysis**

The population was divided into two groups:

- “NIV-failed” group: patients performed NIV for more than three hours after ICU admission and required endotracheal intubation for invasive mechanical ventilation;
- “NIV-successful” group: patients performed NIV and never required invasive mechanical ventilation.

Categorical variables are reported as numbers and percentages (%). Continuous data are reported as mean ± standard deviation (SD) or median, first and third quartile [q_1_-q_3_], according to their distribution, analysed by the Shapiro-Wilk test (α=0.05). We reported minimum (min) and maximum (max) values.

For categorical variables, we analysed contingency tables with the Chi-square test (Χ^2^, α= 0.05). For continuous variables and according to distribution, we used a two-tailed Student t-test (α = 0.05) for independent sample, corrected for equal or unequal variance (Welch test), or the Mann-Withney test (α = 0.05). A *p-value < 0.05* was considered statistically significant.

**Results**

**NIV-failed vs NIV-successful group**

Supplementary table 1 reported minimum, maximum values and missing data for all considered variables.

NIV-failed and NIV-successful groups consisted of 44 and 38 patients, respectively. Tables 2, 3 and 4 showed detailed results of statistical comparison between the two groups. The survival rate in NIV-successful group resulted higher than NIV-failed group (94.7% vs 2.3%, *p-value < 0.0001*, see table 2).

Male gender was more frequent in NIV-successful group (89.5% vs 63.6%, *p-value 0.0069*). Moreover, NIV-successful group patients were younger than NIV-failed patients (59.0±12.4 vs 69.4±7.8 years, *p-value<0.0001*) and showed shorter ICU-LOS (5.0 [4.0-8.0] vs 10.0 [7.0-13.0] days, *p-value<0.0001*). Charlson Comorbidity Index resulted lower in NIV-successful group when compared to failed group (2 [1-3] vs 4 [3-4] points, *p-value<0.0001*), with a lower number of patients suffering from COPD (7.9% vs 27.2%, *p-value 0.0236*). No other differences were noted in comorbidities distribution.

CPAP frequency resulted higher in NIV-successful group (26.3% vs 6.8%, *p-value 0.0166*), while when PSV was performed, pressure support resulted higher in NIV-successful group (9.0 [7.0-10.0] vs 6.0 [5.0-10.0] cmH_2_O, *p-value 0.0393*). Data on first arterial blood gas analysis performed in ICU showed only a lower lactate level for the NIV-successful group (1.2 [1.0-1.6] vs 1.5 [1.3-1.9] mmol/L, *p-value 0.0070*).

NIV-successful group showed lower glycemia (*p-value 0.0039*), troponin (*p-value 0.0008*), CK-MB (*p-value 0.0038*), BNP (*p-value 0.0001*), neutrophil-lymphocyte ratio (*p-value 0.0080*), aPTT (*p-value 0.0041*), D-Dimer (*p-value 0.0037*), and PCT (*p-value0.0141*). On the other side, the same group showed higher eGFR (*p-value 0.0104*), albumin (*p-value 0.0051*), potassium (*p-value 0.0013*), calcium (*p-value 0.0443*), ALT (*p-value 0.0016*), Hb (*p-value 0.0327*), fibrinogen (*p-value 0.0166*), and AT III (*p-value 0.0161*). For details see Table 3.

About therapies (Table 3), tocilizumab was administered to 16 patients (19.5%), eculizumab to 2 patients (2.4%), and remdesivir to 6 patients (7.3%), without statistically significant differences between the groups.

According to our temporal criteria, chest CT scan was performed in 58 patients (70.7%): 31 patients in NIV-failed group (70.5%) and 27 patients in NIV-successful group (71.1%). Χ^2^ test showed no statistically significant difference in the frequency of CT scan between the groups (*p-value 0.9530*). Iodinate contrast was administered in 28 patients (48.3%), and pulmonary signs of thromboembolism were showed in 2 patients (7.1%). Chest CT scan in NIV-successful group showed that ground-glass opacity (*p-value 0.0281*), adjacent pleural thickening (*p-value 0.0090*) and subcutaneous emphysema (*p-value 0.0167*) were less frequent than in NIV-failed group. No other differences were noted, and details are provided in Table 4.

**Reference**

1. Charlson ME, Pompei P, Ales KL, MacKenzie CR: A new method of classifying prognostic comorbidity in longitudinal studies: development and validation. J Chronic Dis 1987, 40(5):373-383. 10.1016/0021-9681(87)90171-8.

2. Vincent JL, Moreno R, Takala J, Willatts S, De Mendonca A, Bruining H, Reinhart CK, Suter PM, Thijs LG: The SOFA (Sepsis-related Organ Failure Assessment) score to describe organ dysfunction/failure. On behalf of the Working Group on Sepsis-Related Problems of the European Society of Intensive Care Medicine. Intensive Care Med 1996, 22(7):707-710. 10.1007/BF01709751.

3. Zahorec R: Ratio of neutrophil to lymphocyte counts--rapid and simple parameter of systemic inflammation and stress in critically ill. Bratisl Lek Listy 2001, 102(1):5-14.

4. Xu X, Yu C, Qu J, Zhang L, Jiang S, Huang D, Chen B, Zhang Z, Guan W, Ling Z et al: Imaging and clinical features of patients with 2019 novel coronavirus SARS-CoV-2. Eur J Nucl Med Mol Imaging 2020, 47(5):1275-1280. 10.1007/s00259-020-04735-9.
