# Supplementary material for: Predictor factors for non-invasive mechanical ventilation failure in severe COVID-19 patients in the intensive care unit: a single-center retrospective study
Source: J Anesth Analg Crit Care. 2022 Feb 15;2:10. doi: 10.1186/s44158-022-00038-7 (PMC8853166; doi:10.1186/s44158-022-00038-7)
Supplement: Supplementary file 2 — Additional file 2: Supplementary file 2. [file 44158_2022_38_MOESM2_ESM.docx]

**Supplementary file 2**

**Charlson Comorbidity Index**

The Charlson Comorbidity Index predicts 10-year survival in patients with multiple comorbidities. The variables considered for its computation are reported in the table.

| **Variable** | **Definition** | **Points** |
| --- | --- | --- |
| Myocardial infarction | History of definite or probable MI (EKG changes and/or enzyme changes) | 1 |
| Congestive heart failure | Exertional or paroxysmal nocturnal dyspnea and has responded to digitalis, diuretics, or afterload reducing agents | 1 |
| Peripheral vascular disease | Intermittent claudication or past bypass for chronic arterial insufficiency, history of gangrene or acute arterial insufficiency, or untreated thoracic or abdominal aneurysm (≥6 cm) | 1 |
| Cerebrovascular accident or transient ischemic attack | History of a cerebrovascular accident with minor or no residua and transient ischemic attacks | 1 |
| Dementia | Chronic cognitive deficit | 1 |
| Chronic obstructive pulmonary disease | - | 1 |
| Connective tissue disease | - | 1 |
| Peptic ulcer disease | Any history of treatment for ulcer disease or history of ulcer bleeding | 1 |
| Mild liver disease | Mild = chronic hepatitis (or cirrhosis without portal hypertension) | 1 |
| Uncomplicated diabetes | - | 1 |
| Hemiplegia | - | 2 |
| Moderate to severe chronic kidney disease | Severe = on dialysis, status post kidney transplant, uremia, moderate = creatinine >3 mg/dL (0.27 mmol/L) | 2 |
| Diabetes with end-organ damage | - | 2 |
| Localized solid tumor | - | 2 |
| Leukemia | - | 2 |
| Lymphoma | - | 2 |
| Moderate to severe liver disease | Severe = cirrhosis and portal hypertension with variceal bleeding history, moderate = cirrhosis and portal hypertension but no variceal bleeding history | 3 |
| Metastatic solid tumor | - | 6 |
| AIDS* | - | 6 |

Plus 1 point for every decade age 50 years and over, maximum 4 points.

Note: liver disease and diabetes inputs are mutually exclusive (e.g. do not give points for both "mild liver disease" and "moderate or severe liver disease").

10-year survival = 0.983^(e^CCI × 0.9^), where CCI = Charlson Comorbidity Index.

**Reference**

- Charlson ME, Pompei P, Ales KL, MacKenzie CR: A New Method of Classifying Prognostic Comorbidity in Longitudinal Studies: Development and Validation. Journal of Chronic Diseases 40:373-383, 1987;
- Quan H, Li B, Couris CM, Fushimi K, Graham P, Hider P, Januel JM, Sundararajan V. Updating and Validating the Charlson Comorbidity Index and Score for Risk Adjustment in Hospital Discharge Abstracts Using Data From 6 Countries. Am. J. Epidemiol. (2011)doi: 10.1093/aje/kwq433;
- Radovanovic D, Seifert B, Urban P, Eberli FR, Rickli H, Bertel O, Puhan MA, Erne P; AMIS Plus Investigators. Validity of Charlson Comorbidity Index in patients hospitalised with acute coronary syndrome. Insights from the nationwide AMIS Plus registry 2002-2012. Heart. 2014 Feb;100(4):288-94. doi: 10.1136/heartjnl-2013-304588. Epub 2013 Nov 1;
- Zavascki AP, Fuchs SC. The need for reappraisal of AIDS score weight of Charlson comorbidity index. J Clin Epidemiol. 2007;60(9):867-8.
