# Supplementary material for: Predictor factors for non-invasive mechanical ventilation failure in severe COVID-19 patients in the intensive care unit: a single-center retrospective study
Source: J Anesth Analg Crit Care. 2022 Feb 15;2:10. doi: 10.1186/s44158-022-00038-7 (PMC8853166; doi:10.1186/s44158-022-00038-7)
Supplement: Supplementary file 3 — Additional file 3: Supplementary Table 1. [file 44158_2022_38_MOESM3_ESM.docx]

| **Minimum, maximum and missing data** | | | |
| --- | --- | --- | --- |
| **Demographic data** | | | |
| **Variable** | **Min** | **Max** | **Missing Data** |
| *Sex Male (%)* | - | - | 0 |
| *Age (years)* | 31.0 | 87.0 | 0 |
| *BMI (kg/m^2^)* | 19.5 | 48.3 | 0 |
| *NIV-day* | 1.0 | 18.0 | 3 |
| *Ward LOS (days)* | 0.0 | 68.0 | 0 |
| *ICU LOS (days)* | 3.0 | 32.0 | 0 |
| *Survived (%)* | - | - | 0 |
| **Comorbidities** | | | |
| **Variable** | **Min** | **Max** | **Missing data** |
| *Charlson Comorbidity Index* | 0 | 8 | 0 |
| *Hypertension (%)* | - | - | 0 |
| *Obesity (%)* | - | - | 0 |
| *Diabets (%)* | - | - | 0 |
| *COPD (%)* | - | - | 0 |
| *CAD (%)* | - | - | 0 |
| *CKD (%)* | - | - | 0 |
| *Endocrinological disease (%)* | - | - | 0 |
| *Atrial Fibrillation (%)* | - | - | 0 |
| *DVT (%)* | - | - | 0 |
| *CVD (%)* | - | - | 0 |
| *Asthma (%)* | - | - | 0 |
| *Autoimmune disease (%)* | - | - | 0 |
| *Haematological disease (%)* | - | - | 0 |
| *OSAS (%)* | - | - | 0 |
| *Neurological disorder (%)* | - | - | 0 |
| *Liver disease (%)* | - | - | 0 |
| **Arterial Blood Gas Analysis and ventilation parameters** | | | |
| **Variable** | **Min** | **Max** | **Missing data** |
| *pH* | 7.25 | 7.55 | 18 |
| *PaO_2_ (mmHg)* | 56.0 | 359.0 |  |
| *PaCO_2_ (mmHg)* | 22.0 | 67.0 |  |
| *PaO_2_/FiO_2_* | 62.0 | 449.0 |  |
| *Lactate (mmol/L)* | 0.5 | 3.5 | 19 |
| *CPAP (%)* | - | - | 0 |
| *PSV (%)* | - | - |  |
| *PEEP (cmH_2_O)* | 6 | 14 | 28 |
| *Pressure Support (cmH_2_O)* | 3.0 | 19.0 | 36 |
| **Laboratory data** | | | |
| **Variable** | **Min** | **Max** | **Missing data** |
| *Glycemia (mg/dL)* | 48.0 | 461.0 | 0 |
| *Azotemia (mg/dL)* | 13.0 | 166.0 | 0 |
| *Creatinine (mg/dL)* | 0.37 | 3.50 | 0 |
| *eGFR (mL/min)* | 15.2 | 135.0 | 0 |
| *Total protein (g/dL)* | 4.5 | 7.3 | 34 |
| *Bilirubin (mg/dL)* | 0.3 | 7.9 | 0 |
| *Albumin (g/dL)* | 2.1 | 4.0 | 0 |
| *Ammonium (µg/dL)* | 36.0 | 178.0 | 21 |
| *Sodium (mEq/L)* | 128.0 | 165.0 | 0 |
| *Potassium (mEq/L)* | 3.1 | 5.7 | 0 |
| *Clorum (mEq/L)* | 93.0 | 123.0 | 27 |
| *Calcium (mg/dL)* | 7.4 | 11.7 | 1 |
| *Magnesium (mg/dL)* | 1.5 | 3.2 | 6 |
| *AST (U/L)* | 15.0 | 157.0 | 0 |
| *ALT (U/L)* | 10.0 | 247.0 | 0 |
| *LDH (U/L)* | 165.0 | 1119.0 | 0 |
| *CPK (U/L)* | 12.0 | 3448.0 | 0 |
| *Troponin (ng/L)* | 1.6 | 3548.0 | 1 |
| *Myoglobin (ng/mL)* | 11.4 | 1604.5 | 2 |
| *CK-MB (ng/mL)* | 0.4 | 28.2 | 4 |
| *BNP (pg/mL)* | 7.0 | 951.0 | 6 |
| *Hb (g/dL)* | 8.5 | 16.3 | 0 |
| *WBC (x10^3^/mm^3^)* | 2.1 | 27.3 | 0 |
| *Neutrophils (x10^3^/mm^3^)* | 1.5 | 25.1 | 0 |
| *Lymphocytes* | 65 | 1749 | 0 |
| *Eosinophils* | 0 | 461 | 0 |
| *Monocytes* | 24 | 1495 | 0 |
| *Basophils* | 0 | 273 | 0 |
| *Neutrophil/Lymphocyte ratio* | 1.5 | 55.8 | 0 |
| *Platelet (x10^3^/mm^3^)* | 20.0 | 520.0 | 0 |
| *aPTT (sec)* | 21.6 | 81.0 | 0 |
| *INR* | 0.93 | 2.74 | 0 |
| *Firbinogen (mg/dL)* | 198.0 | 1055.0 | 0 |
| *D-Dimer (ng/mL)* | 20.0 | 20000.0 | 1 |
| *AT (%)* | 37.0 | 122.0 | 1 |
| *CRP (mg/dL)* | 0.1 | 256.4 | 14 |
| *PCT (ng/mL)* | 0.03 | 44.7 | 21 |
| *SOFA score* | 0 | 6 | 20 |
